# From complexity to clarity: how directed acyclic graphs enhance the study design of systematic reviews and meta-analyses

**DOI:** 10.1007/s10654-023-01042-z

**Published:** 2023-08-31

**Authors:** Stijntje W. Dijk, Lisa M. Caulley, Myriam Hunink, Jeremy Labrecque

**Affiliations:** 1 Department of Epidemiology, Erasmus MC University Medical Center, P.O. Box 2040, 3000CA Rotterdam, The Netherlands; 2 Department of Radiology and Nuclear Medicine, Erasmus MC University Medical Center, Rotterdam, The Netherlands; 3 Department of Otolaryngology-Head and Neck Surgery, University of Ottawa, Ottawa, Canada; 4 Department of Clinical Medicine, Aarhus University, Aarhus, Denmark; 5 Centre for Health Decision Science, Harvard T.H. Chan School of Public Health, Boston, USA

**Keywords:** Research design, Meta-analysis as topic, Causality, Epidemiologic factors, Epidemiologic methods

## Abstract

While frameworks to systematically assess bias in systematic reviews and meta-analyses (SRMAs) and frameworks on causal inference are well established, they are less frequently integrated beyond the data analysis stages. This paper proposes the use of Directed Acyclic Graphs (DAGs) in the design stage of SRMAs. We hypothesize that DAGs created and registered a priori can offer a useful approach to more effective and efficient evidence synthesis. DAGs provide a visual representation of the complex assumed relationships between variables within and beyond individual studies prior to data analysis, facilitating discussion among researchers, guiding data analysis, and may lead to more targeted inclusion criteria or set of data extraction items. We illustrate this argument through both experimental and observational case examples.

## Introduction

Systematic Reviews and Meta-Analyses (SRMAs) are essential tools for evidence-based decision making in many fields. SRMAs that seek to answer causal questions synthesize the evidence across different studies in a systematic manner to minimize bias and provide a quantitative and qualitative appraisal of the evidence. However, these comprehensive evidence summaries can be time-consuming, and their effectiveness is dependent on the quality of the included studies and the interpretation of pooled results.

Causal inference seeks to answer causal questions and limit potential biases [1]. A commonly used tool from the causal inference framework used in study design is the Directed Acyclic Graph (DAG). At its simplest, a DAG is a representation of the data generating mechanism. In other words, it is a visualization composed of nodes (representing the variables) and edges (indicating the direction of causal relationships) that shows the causal relationships between all variables relevant to a given research question or analysis. They are acyclic because they cannot contain cycles wherein one could start at a given node, follow the edges in the direction indicated and end up back at the original node [1].

Sources of bias in a DAG can be found in open backdoor paths. A backdoor path is any path from the exposure to the outcome with an edge that points into the exposure that does not contain a variable that is adjusted for or a collider that is not adjusted for. If the true DAG were known, a researcher would have perfect knowledge of what variables must be adjusted for. Because we cannot know the true DAG, we must instead rely on substantive knowledge of our research question to draw the DAG. This DAG can be used to decide which variables must be adjusted for (and not adjusted for) in an analysis keeping in mind that these decisions are only correct when the DAG itself is correct. In this way, DAGs help investigators visualize their assumptions of causal relationships between the exposure, outcome of interest and covariates [1–3]. We invite readers who are interested in familiarizing themselves further with DAGs and Causal inference consult works such as those by Hernan, Shrier and Digitale [1, 2, 4].

Although frameworks for conducting SRMAs and causal inference are well established, they are less frequently integrated [5]. Some papers describe the use of causal inference techniques during the risk of bias (ROB) assessment [6–9] or the data analysis [7, 8, 10] however we did not identify published examples where the principles of causal inference are explicitly described in the overall design of SRMAs including the construction of the search. In this paper we propose using DAGs at the design stage of SRMAs to ensure an efficient and effective review and illustrate this with both an experimental and observational example.

## Similarities to and differences with common SRMA tools

There are several tools that are commonly applied to conduct systematic reviews in a manner that is transparent, reproducible, specific and detects key sources of bias. A non-exhaustive overview of some of these key tools and the domains they address is presented in Table 1.

**Table 1:** Non-exhaustive list of existing commonly applied tools that help to improve the effectiveness and efficiency of systematic reviews and meta-analyses

Purpose	Tool	Intended effect	Description
Specification of search strategy	PICOS statement	Specify and clarify search criteria and eligibility criteria to guide the search based on the research question	Authors specify the:
			Patient population of interest
			Intervention/exposure
			Comparator/control
			Outcomes of interest
			Study types eligible for inclusion
Pre-registration study protocol	For example: PROSPERO	Reduce bias caused by changes authors make based on findings Increase transparency	Registration in public database of study protocol. Contains items including:
			Search strategy,
			PICOS statement,
			Context/setting,
			Data extraction items,
			Planned risk of bias assessment,
			Strategy for evidence synthesis
	PRISMA-P	Guide reporting by providing a checklist for preferred reporting items for systematic reviews and meta-analysis protocols	Authors specify the
			Funding and support sources and roles
			Rationale and objectives (PICO)
			Eligibility criteria
			Information sources
			Search strategy
			Study records (data management, selection process, data collection process
			Data items
			Outcomes and prioritization
			Risk of bias in individual studies
			Data synthesis
			Meta-bias(es)
			Confidence in cumulative evidence (such as GRADE)
Risk of Bias assessment	ROB2	Identify potential sources and level of risk of bias in RCTs	Identify risk of bias in the following domains and categorize these as low, some concerns, or high risk of bias
			1: bias from randomization process,
			2: bias due to deviations from intended interventions,
			3: bias due to missing outcome data,
			4: bias in measurement of the outcome,
			5: bias in the selection of the reported result
	ROBINS-I	Identify potential sources and level of risk of bias in non-randomized studies of interventions	Identify risk of bias in the following domains and categorize these as low, some concerns, or high risk of bias
			1: pre-/at-intervention: participant selection, classification of interventions,
			2: post-intervention: missing data, bias in measurement of outcomes, bias in selection of reported result
	QUADAS-2	Identify potential sources and level of risk of bias in diagnostic accuracy studies	Specify the relevant:
			Population (presentation and prior test),
			Index test,
			Target condition,
			Reference standard
			Identify risk of bias in the following domains and categorize these as low, some concerns, or high risk of bias
			Patient selection (consecutive or randomized, case control, exclusions),
			Index test (blinding to reference standard result, pre specified cutoff),
			Reference standard (likely to correctly classify disease, blinded to index test result),
			Flow and timing (appropriate time interval, all participants receive same reference standard)

Both DAGs and ROB tools are grounded in the principles of causal inference, which aim to improve the validity and reliability of study findings by identifying potential bias, controlling for confounding, and reviewing the comparability of the study population. However, while ROB tools mainly provide information on suspected bias in individual included studies, DAGs can offer transparent and structured information about the relationships between variables that may not be captured otherwise. This includes the complexity of relationships, potential confounders, and selection bias.

## How DAGs can improve the effectiveness of SRMAs

The effectiveness of SRMA’s refers to their ability to provide accurate and reliable answers to research questions. In addition to current risk of bias tools that can help evaluate the quality of individual studies, DAGs offer a useful approach to improving the effectiveness of SRMAs.

First and foremost, DAGs help provide a visual representation of the relationships between variables in SRMAs, making it easier to understand the complexity of these relationships[1, 2] and identify potential confounding variables beyond the bias of individual studies. The improved transparency allows researchers to communicate their assumptions about the relationships between variables in the analysis as well as potential limitations of the SRMA.

Secondly, DAGs can help guide data analysis by visualizing which variables play specific roles, such as exposure, mediator, and confounder, and create protocols to address them in an appropriate manner. Furthermore, DAGs provide a template that can be used to compare adjustment strategies in different included papers or make decisions to exclude papers on this basis if such biases cannot be addressed in the meta-analyses (for example, if individual-level data would be required).

Lastly, by identifying potential causal relationships that have not been investigated, these DAGs also have the potential to inform further research.

## How DAGs can improve SRMA efficiency

The process of conducting an SRMA can be long and tedious. Efficiency is therefore an important consideration as it enables researchers to produce accurate and reliable results while minimizing the use of resources such as personnel time. While effectiveness must always be preserved to ensure that research questions must be answered accurately and reliably, DAGs can help support the efficiency of SRMAs in several ways.

Firstly, they can help to simplify and limit the number of data extraction items based on their visualization. This reduces the risk of extracting information that would not affect the analysis. Additional aspects of the research question may still be of interest to explore but may not be prioritized. Alternatively, identifying all variables that are required to close all open backdoor paths (non-causal paths from the exposure to the outcome [1]) may also prevent initially missing relevant data extraction items and having to review all papers again.

Secondly, with careful consideration, DAGs could facilitate a narrower selection of studies based on inclusion criteria that are guided by the DAG. Meta-analyses with fewer but high-quality studies that do not apply inappropriate (non-)adjustments of included variables can improve efficiency while maintaining accuracy.

Finally, DAGs can facilitate collaboration between researchers during discussions. Their visual nature can help to communicate complex relationships between variables, leading to a better understanding and discussion of underlying assumptions.

## Two examples

To investigate how DAGs may have led to different approaches in the pursuit of SRMAs, we provide two examples in Table 2. The first example considers a question exploring the causal effect of mindfulness-based interventions on perceived stress in medical students. The aim of the second example is to examine the impact of maternal smoking on the birthweight of newborn children.

**Table 2:** Two examples of research questions and how drawing a DAG could have aided us in our decisions on study design

Research question	What is the effect of mindfulness-based interventions on medical students’ perceived stress?	What is the effect of maternal smoking on birthweight in newborns?
Population, intervention or exposure, comparator, outcome, study type	P: Medical students I: Mindfulness-based Interventions C: No mindfulness-based intervention O: Perceived Stress S: Randomized Controlled Trials	P: Newborns E: Maternal smoking C: No Maternal Smoking O: Birthweight S: Observational studies
Potential DAG	*	
How our DAG may have aided our study design	We see that due to randomization the backdoor path from the treatment assignment to factors L to the outcome is normally closed, meaning we do not require adjustment. However, when the study has loss to follow up, we are conditioning on only the students for whom we observe follow-up, which opens the backdoor path by conditioning on C. If for example students lost to follow up have different stress levels than those who remain, this could introduce bias. To increase effectiveness, we could choose to only include studies that have near complete follow up or that accurately deal with loss to follow-up As it is difficult to blind students to whether they are in the control or the mindfulness-based intervention, this opens additional potential sources of bias. We could decide to perform a sensitivity analysis that explores the impact of only including blinded studies in our meta-analysis In non-randomized trials, there would be an arrow between factors L, such as age/sex/motivation/etc., and treatment. That is because we expect these factors to influence a persons’ choice of doing mindfulness-based interventions spontaneously, but we also think these factors influence their perceived stress. We assume we won’t have access to all potential influencing factors we deem relevant. We therefore decide to exclude studies that are non-randomized. We also show that we assume students’ gained attention control and cognitive reappraisal act as a mediator, which lies on the causal pathway between the exposure and outcome that fully explains the relationship. We therefore decide not to collect data on this parameter	Given our research question we will, due to ethical concerns, likely need to search for observational cohort studies When preparing for our SRMA we notice several studies adjusted for Gestational Age. We draw a DAG to visualize this relationship. Babies that are born earlier have a lower birthweight (arrow G–Y). If smoking causes babies to be born earlier (E–G), you are taking away part of the effect of smoking, ie. the part that is mediated through Gestational Age. We therefore choose to exclude papers that use Gestational Age as a confounder for this study (note that adjustments for G may be appropriate in other research questions) By adding an arrow between smoking and reported smoking (E*), we illustrate to our audience that we expect that there will be measurement error in our study, as individuals tend to provide incomplete information about behaviors that are typically perceived as detrimental to health We have additionally identified several potential confounders including SES, co-morbidity and age that could affect the relationship between smoking and birthweight, we therefore decide to include these items in our data extraction sheet and/or take note of how individual studies have addressed them

*Figure based on Lu et al. [11]

### Discussion of limitations

The use of DAGs in study design is not a new concept, however, to our knowledge, this is the first time they are explicitly proposed in the design of SRMAs the data analysis plan or the evaluation of individual studies. Our paper illustrates how DAGs could potentially be a valuable tool to provide greater transparency and answer meaningful questions more accurately and reliably. We hypothesize that their value may be especially important when used prior to the data collection process. They can visualize assumed causal relationships, aiming to aid both the researcher as well as the audience in their understanding and communication of what needs to be true about the way the data were generated for the analysis to be unbiased. DAGs may help optimize the literature search and evidence synthesis beyond using existing risk of bias tools by using hypothesized relationships between variables to guide choices in choices in study selection, data extraction and data synthesis/analysis.

Despite these hypothesized strengths, some researchers may argue that DAGs are nothing more than common-sense thinking, and that they are not needed when a research protocol is properly defined and pre-registered. However, we contend that even with a well-defined and pre-registered protocol, DAGs still make the underlying knowledge and assumptions more explicit. Another argument made against the use of DAGs in study design is that compared to illustrative examples of DAGs, real-life DAGs can become unwieldy through endless nodes and edges, making them unreadable or difficult to compute all potential consequences. Nevertheless, failing to consider the role all variables play in the causal pathway (from confounder to collider) may lead to biased estimates. Therefore, while DAGs are not perfect, they are still better than the alternative of not having them at all. This principle also applies to situations where insufficient information is available on a particular topic to draw a DAG we are confident in. An imperfect DAG, or multiple possible hypothesized DAGs, then help illustrate the assumptions the researchers are considering. They leave room for the reader to agree or disagree, or guide new analyses when more information becomes available.

Moreover, whereas DAGs form a visual representation of the assumed underlying structure, it must not be forgotten that the DAG is based on assumptions. An incorrectly drawn DAG may give the writer an unfounded sense of confidence in their analysis. While the DAG is only helpful to the analysis if it represents the true underlying causal structure, it can still provide a helpful tool for readers to discuss why they disagree with research findings as the underlying assumptions have been made visually explicit.

While the application of DAGs to mitigate bias in analyses offers several strengths, it is important to recognize that their effectiveness heavily relies on the availability of high-quality and well-reported data. In practice however, primary studies often do not provide the level of detail required for implementing the optimal adjustments suggested by the DAGs. Nevertheless, highlighting the importance of this information gap or the absence of essential parameters and analysis information can serve as a valuable signal for future research to expand or refocus the parameters of interest and promoting higher levels of reporting.

Furthermore, while the suggested uses of DAGs in this paper are argued to increase efficiency and effectiveness of SRMAs, there is a risk that the scope of the analysis becomes too narrow. Narrowing the number of extraction items would be less appropriate for exploratory reviews or when the evidence base for constructing the DAG is less extensive. Researchers should be especially careful when using this efficiency criterion to exclude papers based on what they judge to be low quality. Alternative methods to approach low-quality research, such as weighting, performing sensitivity analyses, or only using information in the qualitative review description may be more appropriate in some settings. When choices were made in order to improve efficiency, researchers should still remain open to the possibility of modifying the DAG when the need becomes apparent during the data collection process.

Additionally, DAGs are not able to visualize all aspects of relationships between variables. They are not designed to visualize effect modification, which occurs when the magnitude or direction of a causal effect is modified by the value of another variable. This is because DAGs are non-parametric and effect modification is not a direct causal relationship between variables, but rather a modification of a relationship. For the same reason, DAGs do not provide information about the strength of relationships, or non-linear relationships.

Another potential limitation is that a specific DAG may not be universally applicable and may have different implications for different populations even when the research question is the same. While the generalizability of the SRMA may be limited in such situations, this does not diminish the overall usefulness of DAGs. Within one study, it is also possible to prepare separate DAGs for subgroups or secondary analyses that reflect different populations to increase the applicability of the SRMAs findings in these populations.

Lastly, while DAGs can help researchers to identify potential confounding variables and control for them in their analysis, they cannot account for unmeasured confounding that may influence the outcome of interest.

## Conclusion

Overall, DAGs are a powerful tool for visualizing the causal structure between variables that generate the data used in SRMAs. By making DAGs, in combination with other SRMA design tools and techniques, an explicit part of the registered study protocol, researchers can ensure that their study design is as rigorous and transparent as possible, ultimately leading to more robust and reliable research findings.
